# Exploring feasible ways of person-reported outcome measurement in routine type 1 diabetes care: a protocol for the Diabeter-PROM study

**DOI:** 10.3389/fcdhc.2026.1729632

**Published:** 2026-02-16

**Authors:** Per Winterdijk, Pim Dekker, Christine Fransman, Erwin Birnie, Henk-Jan Aanstoot, Giesje Nefs

**Affiliations:** 1 Diabeter, Center for Pediatric and Adult Diabetes Care and Research, Rotterdam, Netherlands; 2 Department of Genetics, University Medical Center Groningen, University of Groningen, Groningen, Netherlands; 3 Diabeter Centrum Amsterdam, Amsterdam, Netherlands; 4 Department of Medical Psychology, Radboud University Medical Center, Nijmegen, Netherlands; 5 Department of Medical and Clinical Psychology, Center of Research on Psychological Disorders and Somatic Diseases (CoRPS), Tilburg University, Tilburg, Netherlands

**Keywords:** diabetes care, PROM, psychology, shared decision making, type 1 diabetes

## Abstract

**Background:**

Psychosocial person-reported outcome measurements (PROMs) may assist monitoring, screening and support in routine care for people with type 1 diabetes (PWDs). However, recommended PROM sets are either too limited in covered domains, too time-consuming, or too general to fit type 1 diabetes (T1D) clinical practice. We propose an alternative approach to PROM assessment in routine daily T1D care (Diabeter-PROM study) that is maximally relevant and minimally burdensome for PWDs and healthcare professionals (HCPs). We identify single index questions indicating the need for more in-depth assessment, thereby aligning total assessment length more closely with individual needs.

**Methods:**

Scientist-practitioners identified twelve key psychosocial domains based on literature review, clinical experience and discussion with PWDs: general quality of life, mood, anxiety, diabetes-specific worries, impact, disturbed eating behaviour, self-efficacy, self-esteem, social support/interaction, resiliency, stigma and satisfaction with care. These domains will be divided over three observational mixed-method sub-studies. Through purposive-sampled interviews and panels, HCPs and PWDs (> 10 per sub study) co-determine candidate index questions for each domain. These are then included in three separate cross sectional questionnaire batteries with existing PROMs. Each will be completed by ≥200 PWDs from our clinic. Per domain, the optimal index question and most informative in-depth item set are determined using statistics (test characteristics, item response) and clinical interpretation.

**Discussion:**

We describe the exploration of an alternative way of PROM assessments, adapted to use in regular T1D care. After determining the most suitable index questions, we will apply this system in our daily diabetes practice, taking end-user needs into account and facilitating sustainable PROM implementation in routine care.

## 1 Introduction

Type 1 diabetes (T1D) treatment focuses on achieving normoglycemia to prevent acute and long-term complications. This relies on intensive self-management, including but not limited to frequent insulin administration and glucose monitoring (1). Continuous diabetes self-management is very time consuming, taking on average 4 to 5 hours a day for persons with T1D (PWDs) and/or their care partners (2) and impacts PWDs’ quality of life (QoL) and regular aspects of daily life (e.g. school, work, relations) (1, 3). Recent technological advancements such as automated insulin delivery (AID) can partially automate glucose management, decreasing time spent (4, 5), but access to AID systems is still limited in many countries (6). Despite technological developments, outcome data show that only 30-50% of PWDs reach recommended glucose levels (3, 7). Furthermore, PWDs still report diabetes-specific worries such as fear of hypoglycemia or long-term complications, as well as lack of understanding and limited support from parents, partners, friends and healthcare professionals (HCPs) (8, 9). PWDs also experience more general stress, anxiety, and depression than people from the general population (8, 10, 11). These psychosocial issues negatively affect self-management and daily life issues (12). In daily diabetes care, self-management has largely been based on a one-size-fits-all concept (13) with focus on biomedical outcomes and continuous education (14, 15). Recently, the focus has shifted to individualized care beyond biomedical metrics, including increased awareness of the psychosocial impact of T1D and the need for psychological screening and support (16–20). Therefore, guidelines recommend the assessment of emotional health and QoL in regular daily care (21).

Person-Reported Outcomes (PROs) are outcomes, self-reported by the person concerned, regarding their health, well-being, experiences and effects of their medical condition and treatment. They can be assessed using PRO measures (PROMs), usually questionnaires. PROMs are often used for research purposes (22, 23), population analyses, policy and advocacy (23–25). Applying PROMs in clinical practice can add significant value and information to the individual healthcare process, thereby helping to identify issues and problems hindering optimal glucometrics and quality of life. Several studies show that during implementation of PROMs they were assessed positively by the PWDs, improving communication about their diabetes during consultations. PWDs felt that they and their HCPs were more involved in their treatment and that their needs were more easily discussed (26–30). PROs represent ‘outcomes that matter to people’, an essential part of value-based healthcare (VBHC) systems (31). Therefore, various stakeholders are increasingly requiring HCPs to include PROMs in the assessment of routine care, to monitor and improve care and QoL. The American Diabetes Association (ADA) and the European Association for the Study of Diabetes (EASD) state that HCPs should individualize therapy based on, amongst others, psychosocial factors like emotional well-being and treatment burden (21). PROMs are a suitable tool for this (19), but few PROM initiatives have made it to a broad and firm implementation in daily clinical practice. While there are efforts for standardization of PROs in both research and clinical practice (17, 23), the latter often demands sensitivity to disease-specific and contextual variables like socio-demographic and organizational factors (16, 17, 32). For example, while generic PROMs applicable across conditions enable comparing research findings and population analyses (24), they may be of limited value to T1D care and outcomes. In contrast, diabetes-specific PROMs are better suited to providing detailed insights, needed to improve individual outcomes and QoL, and to personalize care processes (33–35). Furthermore, PROs developed for research often include many items per domain, limiting the number of domains that can be covered, while clinical practice benefits from covering a broad scope of domains with a concise item set. To integrate PROM systems into the consulting room in a feasible way and to ensure their value for both PWDs and HCPs, we need to ensure that all relevant domains are evaluated, the total number of questions is not overwhelming, and each domain is discussed in a shared consultation shortly after completing the questionnaires. Repeating the process yearly would bring PROM use/psychosocial assessment into the real world of clinical care (36).

**Table 1** lists the characteristics of existing diabetes-specific PROM sets. As most of these systems are not T1D-specific, limited in covered domains, too time-consuming or do not follow up on identified issues (15, 37–39), our overarching goal is the development and implementation of a PRO system that can be used in routine clinical T1D care in our clinic. This will strengthen shared decision making (SDM) discussions between PWDs and their HCPs as part of our care program.

**Table 1:** Characteristics of existing diabetes-specific PROM-sets in the adult care context.

Name of PROM set	Publication	Number of domains psychosocial PROMs/other	Type diabetes	Approach to limit number of questions in PROM set: Introduction question	Online survey-system
ICHOM	Nano et al., 2020 (17)	3	T1D & T2D	No	N
DiaPROM	Hernar et al., 2021 (15)	3	T1D	No	Y
DiaFlex	Jensen etal. 2023 (28)	3 PROM/3 treatment specific	T1D	No	Y
Digital Diabetes Q	Eeg-Olofsson et al., 2020 (29)	5 PROMs/2 PREMs	T1D & T2D	No	Y
H2O (diabetes)	Hamilton et al., 2024 (45)	7	T1D & T2D	No	Y
DiaProfil (19)	Skovlund et al., 2019 (19)	10	T1D & T2D	Yes, with an open question	Y
Diabeter PROM		12	T1D	Yes, with multiple choice and follow up in-depth questions	Y

H2O, Health Outcomes Observatory project; ICHOM, International Consortium for Health Outcomes Measurement; PROM, Person-reported outcome measure; PREMs, Person-experienced outcome measure; T1D, type 1 diabetes; T2D, type 2 diabetes.

The PRO system should be sustainable, easy to use in daily diabetes care by PWDs and HCP teams and add value to care. For use in our clinical setting, this system needs to be T1D specific with short questionnaires for multiple domains, that can be filled in online during or shortly before a visit, with immediate transfer of calculated results in the electronic health record of the PWD. Here we describe the design and methodology of the ‘Diabeter-PROM study’, the first stage in the overall project. This study aims to determine a set of index questions that best predict increased scores on in-depth questionnaires for every included domain. An index question is a newly designed domain-specific question that indicates whether further (in-depth) questions are needed to evaluate the domain. The PWD only needs to fill in the in-depth questions when the answer to the index question warrants further investigation. If this is not necessary, the index question for the next domain is presented. Additionally, we will explore the viability of limiting the number of in-depth questions by determining the most informative in-depth item set. In short, the goal is to optimize the feasibility of a PROM system in routine type1 diabetes care.

## 2 Methods

### 2.1 Setting and study outline

The Diabeter-PROM study is an observational mixed-method study which aims to develop and assess a PRO system that can be routinely used in clinical care of PWDs. In three sub studies (PROM1-3: **Table 2**), both PWDs and HCP teams will be involved in the development of this system (16, 17, 19, 26, 27, 33, 40).

**Table 2:** Domains and existing questionnaires used for developing the Diabeter-PROM system (see **Supplementary Table 1** for references).

Domain	Study phase	Tools	Total number of items
1. Quality of life	NA^a^	• QualiMeter^a^ (2 items)	2
2. Mood	PROM1	• World Health Organisation- Five (WHO-5: 5 items) • Patient Health Questionnaire 9 (PHQ-9: 9 items)	14
3.Anxiety	PROM1	• General Anxiety Disorder 7 (GAD-7: 7 items)	7
4. Worries	PROM1	• Problem Areas In Diabetes 20 (PAID-20: 20 items) • Fear of Injecting and Self-testing Questionnaire (D-FISQ: 15 items) • Hypoglycemia Fear Survey-II (HFS-II subscale worries: 18 items) • Hyperglycemia Avoidance Scale (HAS subscale worries: 11 items)	64
5. Impact	PROM2	• Dawn Impact of Diabetes Profile version Diabeter (DIDPvD: 11 items)^b^ • MIND Youth Questionnaire (MY-Q subscale impact & coping: items 2, 3, 4, 7, 14-18, 28)	21
6. Disturbed Eating Behaviour	PROM2	• Diabetes Eating Problem Survey-Revised (DEPS-R: 16 items) • MY-Q (subscale food: items 24–27) • Diabetes Psychosocial Assessment Tool (DPAT subscale weight shape and eating: 3 items)	23
7. Diabetes Stigma	PROM2	Type 1 Diabetes Stigma Assessment Scale (DSAS-1: 19 items) Type 1 Diabetes Distress Scale (T1-DDS negative social perceptions subscale: 4 items)	23
8. Treatment Satisfaction	PROM2	MY-Q (quality of care subscale: 3 items) DQOL adult version (treatment subscale: items 1, 2 and 4) T1-DDS (physician distress subscale: 4 items) Diabetes Management Experiences Questionnaire (DME-Q: 22 items)	32
9. Self-Efficacy	PROM3	• Self-Efficacy for Diabetes Management (SEDM: 10 items) • Confidence In Diabetes Self-care Scale (CIDS: items 1, 7, 8, 12, 13, 14, 16, 17, 19) • Diabetes Empowerment Scale Short form 8 (DES SF-8: 8 items)	27
10. Self Esteem	PROM3	• Kind Lebensqualität (KINDL self-image and self-esteem subscales: 4 items) • My-Q (self-esteem subscale: 1 item) • Rosenberg Self-Esteem Scale (10 items)	15
11. Social Support & Social Interaction	PROM3	• Berlin Social Support Scales (BSSS: 17 items) • My-Q (social interaction subscale: items 3, 4, 9, 10, 11) • Type 1 Diabetes Distress Scale (T1-DDS friends and family distress subscale: items 6, 11, 17, 20) • DPAT (social support for life in general subscale; items 1 and 2)	28
12. Resiliency	PROM3	• Connor-Davidson Resilience Scale (CD-RISC: 10 items)	10

a The QualiMeter was developed for assessing PWDs’ quality of life using two questions, applying a different methodology than described in the present manuscript. Results on use of this instrument will be described elsewhere.

b We added 2 items to the original 7-item DIDP (sleep and exercise).

**Figure 1:**
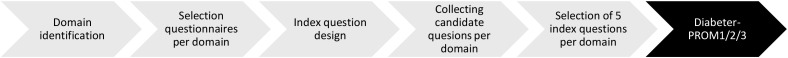
Flowchart diabeter-PROM study.

Using cross-sectional surveys, the index question that best predicts an elevated domain score will be determined (19). Participants will complete the online surveys at home. PROM1, assessing the domains mood, anxiety and diabetes-specific concerns, was conducted in January 2022. PROM2, assessing the domains impact, disturbed eating behaviors, stigma, and satisfaction with diabetes care is scheduled to start early 2026. PROM3, assessing the domains self-efficacy, self-esteem, social support & social interaction, and resiliency is scheduled later in 2026. The methodology and process of exploring this alternative PROM approach are described below and illustrated in **Figure 1**.

#### 2.1.1 Identification of domains

As most PRO research initiatives cover only a limited number of problem areas or domains, the project started with assessing which domains are essential to be addressed in T1D care. Based on categorized literature of psychosocial issues that affect diabetes outcomes, the study team (HJA as pediatrician, GN as psychologist, PW as pediatrician and person with lived experience) identified 12 domains (**Table 2**; **Supplementary Table S1**). Three guidance criteria apply (1): does the domain affect diabetes care or outcomes? (2) can the domain be assessed using one or multiple existing questionnaires? (3) is there scope for improvement of PROs (i.e. are support/intervention options available in case of elevated scores)? (**Table 2**). The selected/identified domains of the Diabeter-PROM study were discussed with PWD in individual interviews, with members of the “Diabeter Panel” in panel discussions, with the client council (all PWD) and with staff members of Diabeter (**Table 3**). These interviews and discussions did not lead to major adjustments of the domains.

**Table 3:** Information about interviews, panel discussions and project timelines.

Sub Study	Domain	Individual interviews with PWD^a^	Diabeter panel^b^, all PWD discussion	Panel discussion Client Council (all PWD)	Panel discussion staff: diabetes nurses & dieticians^c^	Panel discussion staff: medical doctors^d^	Discussion timeline: Domains, Interviews & Panel discussions	Data collection timeline
PROM1	Mood	N=6	–	N=5	Y	Y	May – September 2020	January – March 2022
PROM1	Anxiety	N=6	–	N=5	Y	Y	May – September 2020	January – March 2022
PROM1	Worries	N=6	–	N=5	Y	Y	May – September 2020	January – March 2022
PROM2	Impact	N=8	–	N=4	Y	Y	May – October 2023	Q2 2026
PROM2	Disturbed Eating Behavior	N=8	–	N=4	Y	Y	May – October 2023	Q2 2026
PROM2	Diabetes Stigma	N=8	N=20	N=5	Y	Y	May – November 2024	Q2 2026
PROM2	Treatment Satisfaction	N=8	N=20	N=5	Y	Y	May – November 2024	Q2 2026
PROM3	Self Efficacy	N=8	N=20	N=5	Y	Y	May – November 2024	Q1 2027
PROM3	Self Esteem	N=8	N=20	N=5	Y	Y	May – November 2024	Q1 2027
PROM3	Social Suport & Social Interaction	N=8	N=20	N=5	Y	Y	May – November 2024	Q1 2027
PROM3	Resiliency	N=8	N=20	N=5	Y	Y	May – November 2024	Q1 2027

a demographic information interviewees:.

PROM1: 67% women, mean age 24.6 years, mean diabetes duration = 13.9 years

PROM2: 38% female, mean age 29.9 ye, mean diabetes duration = 16.3 ye

PROM3: 38% female, mean age 30.9 ye, mean diabetes duration = 17.3 ye

b Diabeter panel. Held in February 2024 with at present 130 active members (diverse group of persons living with diabetes receiving treatment from a HCP of Diabeter internal medicine and pediatrics alike; some are parents or caregivers of a child living with diabetes).

c Internal education sessions for diabetes nurses and dieticians. Estimated number of people attending and participating in the panel discussion = 20 to 30.

d Internal education sessions for medical physicians (pediatics & internal medicine; N = 10).

#### 2.1.2 Choice of tools per domain: the in-depth questions

For the first domain, QoL, we developed the QualiMeter (General QoL), consisting of a general and a diabetes-specific QoL question (Which number best describes your life in general? 10 = best, 1 = worst, Diabetes QoL: Which number best describes your diabetes lately? 10 = best, 1 = worst). Results on the use and outcomes of this instrument, which we are now implementing at our clinic, will be described elsewhere. For the remaining 11 domains covered in the Diabeter-PROM study, we selected questionnaires (or subscales thereof) that are often used in research and/or clinical care and are free and publicly available. During the selection procedure it was ensured that the range of questions covered the width of the domain and that measures were applicable to T1D (**Table 2**).

#### 2.1.3 Development of a two-step approach

Our approach is inspired by a previous Danish initiative: the PRO Diabetes Questionnaire, embedded in the stand-alone PRO diabetes digital tool DiaProfil. This system uses multiple domains and index questions for each domain, asking whether a PWD would like to examine and discuss this domain (40). Here, not filling in a domain could mean the PWD has no problem with this domain or does have a problem but does not want to discuss the domain. Our goal is to optimize applicability and useability of the Diabeter-PROM system in routine diabetes care, for PWDs, HCPs and the organisation alike. We therefore set out to develop an index question per domain: a newly designed question that reflects the underlying in-depth questionnaires of the domain sufficiently to be able to signify the need of additionally completing them. The use of index questions will ensure that every domain is assessed and, if necessary, evaluated by a set of in-depth questions. In the context of shared decision making, we aim to ask participants about all domains in order to normalize the use of PROs in clinical care and to offer individuals the opportunity to gain new insights through questions they might not have previously considered. In clinical practice, the agenda for the consultation is always jointly determined, allowing PWD the opportunity to not discuss specific topics. Although PWDs appreciate discussing all domains covering their needs, they may be deterred by too many/too lengthy questionnaires (26). Our approach should prevent this risk of questionnaire fatigue and increase personal relevance, while still addressing every domain.

#### 2.1.4 Identification of index questions

For each domain, potential index questions were collected through individual interviews and panel discussions with PWD, the client council and HCPs (**Table 3**). First, the selected questionnaires for every domain were presented to and read by the interviewees (not filled in) who reflected on them verbally, including discussion of legitimacy and applicability. Second, the idea of developing index questions was explained, and the interviewees were asked to formulate potential index questions per domain that would signal elevated scores on the in-depth questions of the specific domain(s). Third, three scientist-practitioners (PW, GN and HJA) selected the top three most potential index questions based on their clinical and research experience, rating them individually on a 1–3 scale. The five candidate index questions with the highest total scores were included for quantitative evaluation in the surveys. The optimal index question for each domain was then determined through statistical analysis (see section 2.6.).

#### 2.1.5 Determining the most informative in-depth questions

Next, multiple existing questionnaires per domain were selected as in-depth questions. For an efficient and easy-to-use PRO system with minimized risk of questionnaire fatigue, the number of in-depth questions per domain ideally would be 5–10 items (41). For each domain, we determined the most informative items (see section 2.6), to explore whether existing questionnaires would allow such a limitation of items.

### 2.2 Ethics approval

This study is conducted in compliance with the principles of the Declaration of Helsinki (2013 revision). Ethical review for the first part of the Diabeter-PROM1 study has already been waived (METC Oost-Nederland: 2021-13162). The same procedure will be followed for PROM2 and PROM3.

### 2.3 Calculation of sample size

We performed a sample size calculation for the identification and validation of the index questions (i.e., not for validation of the final PROM system). We used the sample size calculation for adequate sensitivity/specificity (no hypothesis testing) (42). With a specificity of 0.80, a sensitivity of 0.80, a prevalence of 0.30, assuming a margin of error of 0.10 and a 95% confidence interval, an effective sample size of at least 205 PWDs per PROM substudy is required for the sensitivity and at least 88 PWDs for the specificity. These assumptions and estimations correspond with a negative predictive value of 0.90 (exact Wilson’s 95% confidence interval: 0.86–0.94). With an expected response rate of approximately 20%, all eligible PWD at our clinic will be invited for each PROM substudy.

### 2.4 Participants & recruitment

People aged ≥16 years with T1D who receive routine diabetes care at the Dutch Diabeter clinics are eligible to participate in the three phases of the PROM study. Exclusion criteria are insufficient language skills and circumstances complicating consent or participation (e.g. certain co-morbidities or problems, which are determined at the discretion of the treating diabetes HCPs). For each PROM-study phase all eligible PWDs receive an invitation email containing a link to the participant information folder (PIF), consent for participation, and a link to the study questionnaire after consent is provided. Questionnaire results are stored on a secure clinic server.

### 2.5 Baseline data

Demographic and clinical data are extracted from the participants’ electronic health records. The following demographic and clinical parameters are extracted: gender, age, duration of diabetes, socioeconomic status, type of treatment modality and HbA_1c_ at the time of completing the questionnaire. Consistent with General Data Protection Regulation (GDPR) requirements, data are pseudonymized and the dataset is stored on a secure clinic server. Participants have the right to view their personal data and have it corrected or destroyed.

### 2.6 Planned statistical analysis

Descriptive statistics (frequencies and percentages, mean and standard deviation, median and interquartile range) are used to characterize the sample. For each domain, sensitivity, specificity, positive and negative predictive values, and likelihood ratios with 95% confidence intervals are estimated to determine which of the five candidate index questions best detects PWDs with elevated score(s) on the in-depth questions. We were guided by the highest NPV in determining which index question was selected per domain. Detection of an elevated domain score is based on a combination of existing cut-off values of total scores as well as on clinical relevance of individual item scores (see **Table 4** for an example of this approach for the Mood, Anxiety and Diabetes-specific concerns domains). Methods from item response theory (43) are used to determine if and how the set of in-depth question(naire)s can be shortened for each domain. IRT is a set of psychometric models to describe the relationship between (observable) responses to a test survey and its items, and an underlying (non-observable) construct. Performance of the survey and items are based on the item difficulty, discriminatory ability and, depending on the context, random guessing. For an example, see Grabman et al. (43). After reduction, predictive ability of the candidate index questions is calculated again for each domain, using the shortened item set to describe any changes in precision metrics between the original and shortened versions. Clinical discussion then determines the optimal index question(s) and final set of in-depth question(naire)s for each domain.

**Table 4:** Definition of elevated domain scores, using mood, anxiety and diabetes-specific concerns as examples.

Domain	Definition of elevated domain score
Mood	PHQ-9 total score ≥10 *or* PHQ-9 individual item score 2 or 3 *or* WHO-5 total score ≤ 50 *or* WHO-5 individual item score 0, 1 or 2
Anxiety	GAD-7 total score ≥ 10 *or* GAD-7 individual item score 2 or 3
Diabetes-specific concerns	PAID-20 total score ≥ 17 *or* PAID-20 individual item score 2, 3 or 4 *or* D-FISQ individual item score 1, 2 or 3 *or* HFS-II individual item score 3 or 4 *or* HAS individual item score 3 or 4

D-FISQ, Fear of Injecting and Self-testing Questionnaire; GAD-7, General Anxiety Disorder 7; HAS, Hyperglycemia Avoidance Scale; HFS-II, Hypoglycemia Fear Survey-II; PAID 20, Problem Areas In Diabetes 20; PHQ-9, Patient Health Questionnaire; WHO-5, World Health Organisation- Five.

## 3 Discussion

Use of PROMs in routine clinical practice will contribute to the urgent need for (1) delivering individualized T1D care beyond biomedical metrics (2), increased psychological screening and support and (3) and improvement of diabetes care and QoL of PWDs (16–19). Currently, most existing diabetes PROM systems are not T1D-specific, limited in domains covered, time-consuming and often do not incorporate actions or referrals towards solving the identified issues. However, there are initiatives to develop questionnaire sets for use in routine T1D care and clinical practice (e.g. the DiaFlex Care PROM (28), the DiaPROM pilot trial (15, 44), the digital Diabetes Questionnaire (29), the DiaProfil PROM (19) and the H2O initiative (45) (**Table 1**). We describe the development of an alternative approach to PRO measurement (Diabeter-PROM study), aimed specifically or use in routine T1D care, with the aim to be maximally relevant for PWDs, HCPs and the organisation alike and minimally burdensome for PWDs and HCPs. Part of this study is exploring to what extent existing questionnaires fit this aim. Many existing questionnaires (used for the in-depth assessments in this study) comprise too many questions to be practical for use in daily routine diabetes care. Our suggested strategy of index questions signalling the need for in-depth assessment of a certain domain—validated against elevated scores on existing questionnaires—is designed to help in preventing question overload and questionnaire fatigue, while still covering multiple psychosocial domains. Furthermore, we will also assess if the number of in-depth questions can be limited for each domain, by determining the most informative items per domain (46). If questionnaire copyright holders do not consent to use the resulting abbreviated sets in our clinical practice, alternative (though not preferred) options include using the short-form (SF) versions of existing questionnaires or custom-made question(naire)s for each domain. This approach is similar to the Danish initiative (PRO Diabetes Questionnaire, embedded in the stand-alone PRO diabetes digital tool DiaProfil (40)) which uses introduction questions for each domain. The type of introduction questions used there (“Do you want to discuss this item with your HCP?”) could lead to misinterpreting the results however, because not filling in a domain could mean the PWD has no problem with this domain or does have a problem but does not want to discuss the domain. The Diabeter-PROM system enables us to cover practically all psychosocial domains that have an impact on diabetes outcome and that can be or need to be improved. With the index questions we are sure all domains are checked. Domains that need attention will be investigated further with the in-depth domain questionnaires, focusing on the problems that need attention. All PROM results will be discussed during a yearly shared decision making (SDM) consultation with the PWD individually and follow up treatment will be initiated when needed if desired.

Key strengths of our proposed approach include the extensive combined statistical and clinical assessment of the questions/items included in the system, closely involving the end users throughout the process (9, 27–30). Second, we aim to include more domains than covered by the International Consortium for Health Outcomes Measurement (ICHOM) standard set (17) in the Diabeter PROM-system. The inclusion of more domains, providing optimal coverage of physical and psychological health, has recently been recommended in a consensus statement for diabetes research and in a systematic review of the European Health Outcomes Observatory (22, 45, 47). The third strength is the two-step approach, using index questions to determine whether in depth evaluation is necessary, which decreases workload and supports acceptance by both PWDs and HCPs.

The approach also has potential limitations. First, the exclusion of questionnaires taht are not free and publicly available may affect the optimal sets of in-depth questions. Second, this study currently focuses on psychosocial PROMs without including social structure, educational level and information about the involvement of other HCPs, including mental health or social care. These factors are already integrated in our clinic SDM process, and the PROMs will eventually be added to this process. Third, this study only includes PWDs aged ≥16 years. For younger PWDs an adapted PRO system will need to be developed and tested, covering relevant domains for children and their caregivers. Fourth, although the rigorous scientific evaluation of the PROM system strengthens its credibility for clinical use, the time-intensive nature of this process means that newly developed instruments, such as the T1DDAS (48), cannot always be included in the PRO set immediately. Consequently, the set will require periodic updates to reflect the evolving landscape of available instruments.

*Future plans: Implementation & training*

Organizations will have to make managerial decisions to initiate development of PROM structures. In addition, implementation in clinical workflows can be challenging and varies significantly between organizations (9, 49). It requires a well-developed strategy and preparation. We identify five areas essential for successful implementation of our PRO system.

First, one of the most important facilitating factors in PROM implementation is training of both HCPs and PWDs to explain the purpose of the PROM system, to clarify its value (also addressing attitudes relating to the need to include psychosocial aspects and have a more holistic approach to care) and to use it in their discussions and communication (15, 50) (33). Results of PROMs and interventions always need to be discussed and aligned with the wishes and needs of the PWD. The second requirement for successful implementation of PROMs into routine care is the need for adequate ICT technology, including data flow (19, 32, 51). Results of PROMs must be easily collected and be accessible to both PWDs and HCPs in electronic health records systems and apps, in a sustainable manner, with minimal burden and maximal benefit (32). A comprehensive online display of the PROM data, with information on various interventions and peer reported experiences will not only improve the SDM discussion but can help PWDs and HCPs to choose the optimal personal treatment option. Modern digital platforms allow identification and discussion of a multitude of factors, personalizing PROM results (26).

Third, a care system able to act on identified issues needs to be in place. This includes training team-members in discussing and first responding to issues. To ensure that psychosocial issues are addressed appropriately, referrals to HCPs with other expertise (e.g. psychologist, social worker) or online psychoeducation, need to be part of the standard care system.

Fourth, diabetes care needs to develop from a glucose-centric and education-centred care to personalized medicine that includes individual needs of PWDs (16–19). This will not only involve measurement and support at the level of the individual, but also at the policy level, e.g. improvement of access to care through modifications in legislation and reimbursements. Fifth, being an important part of our VBHC approach, the assessment of the long-term impact of the PROM system will be an essential part of our implementation strategy, not in the least from the PWD point of view (9, 32). To our knowledge, no studies have assessed if PROM systems and subsequent referrals actually improve quality of life and solve issues for PWD. Published data was mainly collected in academic medical settings with different facilitating factors and barriers compared with other settings (32).

## 4 Concluding

There is a need to develop a PROM system that is sustainable, minimally burdensome and maximally benefitting in routine T1D care (32). Before such a system can be implemented it should be rigorously aligned with routine care needs based on scientific foundation. Once implemented, issues identified by the PROM system can be discussed and addressed by the PWD and the HCP team during a consultation or as part of shared decision-making discussion. Our Diabeter-PROM study is designed to result in a feasible and efficient PROM system aimed specifically at PWDs to primarily identify if, what and to what extent PWDs are experiencing psychosocial issues or barriers in their lives with T1D that warrant further discussion and potential support.
