# Supplementary material for: Exploring feasible ways of person-reported outcome measurement in routine type 1 diabetes care: a protocol for the Diabeter-PROM study
Source: Front Clin Diabetes Healthc. 2026 Feb 16;7:1729632. doi: 10.3389/fcdhc.2026.1729632 (PMC12950607; doi:10.3389/fcdhc.2026.1729632)
Supplement: Supplementary file 1 [file Table1.docx]

**Supplemental Table 1.** Domains and existing tools with references.

| **Domain** | **Tools** |
| --- | --- |
| 1. Mood | - World Health Organisation- Five Well-Being Index (WHO-5: 5 items)[1] - Patient Health Questionnaire 9 (PHQ-9: 9 items)[2] |
| 2.Anxiety | - General Anxiety Disorder 7 (GAD-7: 7 items)[3] |
| 3. Worries | - Problem Areas In Diabetes 20 (PAID-20: 20 items)[4] - Fear of Injecting and Self-testing Questionnaire (D-FISQ: 30 items)[5] - Hypoglycemia Fear Survey-II (HFS-II: 18 items)[6] - Hyperglycemia Avoidance Scale (HAS: 20 items)[7] |
| 4. Impact | - Dawn Impact of Diabetes Profile (DIDP: 6 items)[8] - MIND Youth Questionnaire (MY-Q; items 2, 3, 4, 7, 14–18, 28)[9] - HFS-II (coping subscale; 5 items)[6] - Hyperglycemia Avoidance Scale (HAS; 20 items) - Diabetes Quality-Of-Life, adults (DQOL:items 1–5, 8, 10 11, 13, 14, 16, 18, 25, 29, 31, 32, 34, 40, 41, 42)[10] - Pittsburgh Sleep Quality Index (PSQI: items 4, 6, 8, 9) - VVV (items 1, 2, 3, 4)[11] - Female Sexual Distress Scale (FSDS: 12 items)[12] |
| 5. Disturbed Eating Behaviour | - Diabetes Eating Problem Survey-Revised (DEPS-R; 16 items),[13] - Risk Factors for Binge-Eating Disorder in Overweight (REO; 32 items)[14] - Eating Disorders Examination Questionnaire (EDE-Q; items 1-16, 19, 21, 23, 25, 27, 29-36)[15] - MY-Q (subscale food; items 24–27) - Diabetes Psychosocial Assessment Tool weight (DPAT; shape and eating subscale; 3 items)[16] |
| 6. Self Efficacy | - Self-Efficacy for Diabetes Management (SEDM; 10 items)[17] - Confidence In Diabetes Self-care scale (CIDS; items 1, 7, 8, 12, 13, 14, 16, 17, 19)[18] - Diabetes Empowerment Scale Short form 8 (DES SF-8; 8 items)[19] |
| 7. Self Esteem | - KINDL (generic quality of life instrument for children; self-image and self-eteem subscales; 4 items)[20] - My-Q (self-esteem subscale; 1 item) - Rosenberg Self-Esteem scale (10 items)[21] |
| 8. Social Support & social Interaction | - Berlin Social Support Scales (BSSS; 17 items)[22] - My-Q (social interaction subscale; items 3, 4, 9, 10, 11) - Type 1 Diabetes Distress Scale (T1-DDS; friends and family distress scale; items 6, 11, 17, 20)[23] - DPAT (social support for life in general subscale; items 1 and2) |
| 9. Resiliency | - Connor-Davidson Resilience scale (CD-RISC: 10 items)[24] |
| 10. Diabetes Stigma | - Type 1 Diabetes Stigma Assessment Scale (DSAS-1; 19 items)[25] - Type 1 Diabetes Distress Scale (T1-DDS; negative social perceptions subscale; 4 items) |
| 11. Treatment Satisfaction | - MY-Q (quality of care subscale: 3 items) - DQOL Adults (items 1, 2 and 4) - T1-DDS (physician distress subscale; 4 items) - Diabetes management experiences questionnaire (DME- Q : 22 items)[26] |

**References:**

1. Topp CW, Østergaard SD, Søndergaard S, Bech P. The WHO-5 Well-Being Index: a systematic review of the literature. *Psychotherapy and psychosomatics* 2015; **84**:167-176.

2. Kroenke K, Spitzer RL, Williams JB. The PHQ-9: validity of a brief depression severity measure. *Journal of general internal medicine* 2001; **16**:606-613.

3. Spitzer RL, Kroenke K, Williams JB, Löwe B. A brief measure for assessing generalized anxiety disorder: the GAD-7. *Archives of internal medicine* 2006; **166**:1092-1097.

4. Polonsky WH, Anderson BJ, Lohrer PA, Welch G, Jacobson AM, Aponte JE*, et al.* Assessment of diabetes-related distress. *Diabetes care* 1995; **18**:754-760.

5. Snoek FJ, Mollema ED, Heine RJ, Bouter LM, van der Ploeg HM. Development and validation of the diabetes fear of injecting and self-testing questionnaire (D-FISQ): first findings. *Diabetic medicine : a journal of the British Diabetic Association* 1997; **14**:871-876.

6. Gonder-Frederick LA, Schmidt KM, Vajda KA, Greear ML, Singh H, Shepard JA*, et al.* Psychometric properties of the hypoglycemia fear survey-ii for adults with type 1 diabetes. *Diabetes care* 2011; **34**:801-806.

7. Singh H, Gonder-Frederick L, Schmidt K, Ford D, Vajda KA, Hawley J*, et al.* Assessing hyperglycemia avoidance in people with Type 1 diabetes. *Diabetes Management* 2014; **4**:9.

8. Holmes-Truscott E, Skovlund SE, Hendrieckx C, Pouwer F, Peyrot M, Speight J. Assessing the perceived impact of diabetes on quality of life: Psychometric validation of the DAWN2 Impact of Diabetes Profile in the second Diabetes MILES - Australia (MILES-2) survey. *Diabetes research and clinical practice* 2019; **150**:253-263.

9. de Wit M, Winterdijk P, Aanstoot HJ, Anderson B, Danne T, Deeb L*, et al.* Assessing diabetes-related quality of life of youth with type 1 diabetes in routine clinical care: the MIND Youth Questionnaire (MY-Q). *Pediatric diabetes* 2012; **13**:638-646.

10. The DCCT Research Group. Reliability and validity of a diabetes quality-of-life measure for the diabetes control and complications trial (DCCT). *Diabetes care* 1988; **11**:725-732.

11. Alberts M, Smets EM, Vercoulen JH, Garssen B, Bleijenberg G. ['Abbreviated fatigue questionnaire': a practical tool in the classification of fatigue]. *Nederlands tijdschrift voor geneeskunde* 1997; **141**:1526-1530.

12. Derogatis LR, Rosen R, Leiblum S, Burnett A, Heiman J. The Female Sexual Distress Scale (FSDS): initial validation of a standardized scale for assessment of sexually related personal distress in women. *Journal of sex & marital therapy* 2002; **28**:317-330.

13. Wisting L, Wonderlich J, Skrivarhaug T, Dahl-Jørgensen K, Rø Ø. Psychometric properties and factor structure of the diabetes eating problem survey - revised (DEPS-R) among adult males and females with type 1 diabetes. *Journal of eating disorders* 2019; **7**:2.

14. Wever MCM, Dingemans AE, Geerets T, Danner UN. Screening for Binge Eating Disorder in people with obesity. *Obesity research & clinical practice* 2018; **12**:299-306.

15. Luce KH, Crowther JH. The reliability of the Eating Disorder Examination-Self-Report Questionnaire Version (EDE-Q). *The International journal of eating disorders* 1999; **25**:349-351.

16. Bachmeier CAE, Waugh C, Vitanza M, Bowden T, Uhlman C, Hurst C*, et al.* Diabetes care: addressing psychosocial well-being in young adults with a newly developed assessment tool. *Internal medicine journal* 2020; **50**:70-76.

17. Grossman HY, Brink S, Hauser ST. Self-efficacy in adolescent girls and boys with insulin-dependent diabetes mellitus. *Diabetes care* 1987; **10**:324-329.

18. Van Der Ven NC, Weinger K, Yi J, Pouwer F, Adèr H, Van Der Ploeg HM*, et al.* The confidence in diabetes self-care scale: psychometric properties of a new measure of diabetes-specific self-efficacy in Dutch and US patients with type 1 diabetes. *Diabetes care* 2003; **26**:713-718.

19. Anderson RM, Fitzgerald JT, Gruppen LD, Funnell MM, Oh MS. The Diabetes Empowerment Scale-Short Form (DES-SF). *Diabetes care* 2003; **26**:1641-1642.

20. Ravens-Sieberer U, Bullinger M. Assessing health-related quality of life in chronically ill children with the German KINDL: first psychometric and content analytical results. *Quality of life research : an international journal of quality of life aspects of treatment, care and rehabilitation* 1998; **7**:399-407.

21. Rosenberg M. Society and the adolescent self-image. Princeton University Press 1965.

22. Schulz U, Schwarzer R. Long-term effects of spousal support on coping with cancer after surgery. *Journal of Social and Clinical Psychology* 2004; **23**:716-732.

23. Fisher L, Polonsky WH, Hessler DM, Masharani U, Blumer I, Peters AL*, et al.* Understanding the sources of diabetes distress in adults with type 1 diabetes. *Journal of diabetes and its complications* 2015; **29**:572-577.

24. Connor KM, Davidson JR. Development of a new resilience scale: the Connor-Davidson Resilience Scale (CD-RISC). *Depression and anxiety* 2003; **18**:76-82.

25. Browne JL, Ventura AD, Mosely K, Speight J. Measuring Type 1 diabetes stigma: development and validation of the Type 1 Diabetes Stigma Assessment Scale (DSAS-1). *Diabetic medicine : a journal of the British Diabetic Association* 2017; **34**:1773-1782.

26. Hendrieckx C, Husin HM, Russell-Green S, Halliday JA, Lam B, Trawley S*, et al.* The diabetes management experiences questionnaire: Psychometric validation among adults with type 1 diabetes. *Diabetic medicine : a journal of the British Diabetic Association* 2023:e15195.
